# Influence of Acetone and Primer on Strength and Ductility of Chlorinated Poly(vinyl chloride)

**DOI:** 10.3390/polym15030489

**Published:** 2023-01-17

**Authors:** Hanlin Mi, Xiaokang Huang, Pean-Yue Ben Jar

**Affiliations:** Department of Mechanical Engineering, University of Alberta, Edmonton, AB T6G 1H9, Canada

**Keywords:** chlorinated poly(vinyl chloride), strength, ductility, acetone, primer

## Abstract

Primer is widely used to prepare bonding of chlorinated poly(vinyl chloride) (CPVC) pipe. The study examined the influences of primer and its major component, acetone, on CPVC’s mechanical properties. Two types of CPVC product, sheet and pipe, were used in the mechanical testing. Sheet specimens were immersed in acetone or primer for 40 and 10 min, respectively, i.e., the maximum allowable time without mass loss, and then dried in air before the mechanical testing. Pipe (ring) specimens were treated either through immersion in acetone or primer for 30 min or in contact with these solvents locally on the inner surface for 2.5 h, and then air dried for 10.5 days before the mechanical testing. Results showed that CPVC’s strength decreased after the absorption of these solvents, and air dry could remove acetone but not completely primer. The study also showed that pipe specimens by local contact with primer could generate brittle fracture. In view that sheet specimens always fractured in a ductile manner, brittle fracture of the pipe specimens could not be caused by CPVC degradation. Rather, strength decrease in the local region could provide a plausible explanation for the brittle fracture behavior, though further investigation is needed.

## 1. Introduction

A common practice to evaluate polymers for their suitability in engineering applications is to quantify their mechanical properties such as elastic modulus and yield strength using coupon specimens. This approach has also been adopted for investigating the influence of chemical solvents on the mechanical properties, especially for resisting the environmental stress cracking (ESC) e.g., [1,2]. However, due to the long duration required for the specimen treatment in order to saturate the chemical solvents in the specimens, and the possibility of mass loss due to material dissolved in the chemical solvents, specimens used in the mechanical testing are usually not fully saturated with the chemical solvents, nor are the chemical solvents distributed uniformly in the specimens. As a result, clear conclusions could not be drawn from the testing in order to characterize the influence of chemical solvents on ESC resistance of polymers.

This paper is concerned with failure of chlorinated poly(vinyl chloride) (CPVC) pipe caused by the exposure to chemical solvents. Primer and acetone were used as the chemical solvents in the study. The former is a commercial product that has been widely used to clean and soften CPVC pipe surface before joining in a solvent welding process [3], and is a mixture of acetone, methyl ethyl ketone (MEK), cyclohexanone (CYH) and tetrahydrofuran (THF) [4] with acetone as the major component. Although the primer has been used for many years, some people are still concerned about its use in joining CPVC pipes, as some solvents in the primer were suspected to be responsible for the ductility decrease of the pipe [5].

Since primer is used to soften surface layer of CPVC pipe before the joining, primer acts as a plasticizer [6], and thus it has been suspected that the use of primer may introduce ESC in the CPVC pipe. However, the role of primer on the CPVC pipe failures has attracted little attention so far, even though ESC in CPVC pipe has been reported in the literature [7,8].

For acetone, it has been included in a recently updated list of chemicals which could cause cracking during CPVC pipe installation at freezing temperatures [9]. Although such a list is mainly for the purpose of precaution, it also motivated us to clarify the role of acetone on the crack generation in CPVC pipe. Moreover, the lack of specific details for the cause of cracking could lead to uncertainty on the guidance for the proper practice of joining CPVC pipe, resulting in a significant variation in the actual service life of the pipe from the designed service life of 50 years.

It should be noted that in an early study by Yue [10], suitable chemical solvents for solvent welding have been suggested to be the solvents that can dissolve the polymer to form a surface layer of gel of highly mobile molecular chains, and do not need to be rapidly diffused into the polymer. Although plenty of studies have supported such an idea [1,11,12], none of the studies considered possible issues generated by the presence of excessive chemical solvents that could be left on the pipe surface but not later covered by adhesives to bond with a matching surface. At present, it is not clear whether the presence of such a surface could have any adverse effect on the strength and integrity of the bonded pipe assembly. The study presented here was designed to examine the mechanical performance of CPVC that has been in touch with primer or acetone, either to form a soft layer on the surface or to diffuse through the whole thickness, to elucidate the influence of the exposure on the mechanical properties of CPVC.

This study is part of a project for development of a new method to quantify mechanical performance of CPVC pipe joints, especially after the solvent welding process. This paper is to examine the mechanical properties for CPVC and its pipe products after exposure to either primer or acetone, and to compare the mechanical properties with those for virgin specimens, i.e., without any exposure to the chemical solvents.

## 2. Experimental

Experiments conducted in this study consisted of two parts. One was to use thin CPVC sheets to achieve a relatively uniform content of the chemical solvent in the specimens, and the other to use CPVC pipe to mimic its solvent exposure in service. Details of specimen preparation, mechanical testing and post-fracture examination are given as follows.

### 2.1. Materials and Specimen Preparation

Two types of specimens were used for the mechanical testing, dog-bone specimens from thin sheets to shorten the time for thorough absorption of the chemical solvents and to enhance the uniformity of the solvent distribution, and ring specimens from CPVC pipe to mimic the surface contact of CPVC with the chemical solvents in applications. The thin sheets were commercial CPVC products of 1/32-inch thick (about 0.8 mm), purchased from McMaster-Carr. Such thickness was selected so that the specimens could be handled relatively easily for the mechanical testing, but still allowed saturation of the solvents in a reasonably short immersion period. The pipe was commercial CPVC 4120 SCH80 2-inch (~50 mm) pipe, which was the same grade as the one used in a previous study [13], to examine the influence of the chemical solvents on strength and ductility in the hoop direction of the pipe.

**Figure 1:**
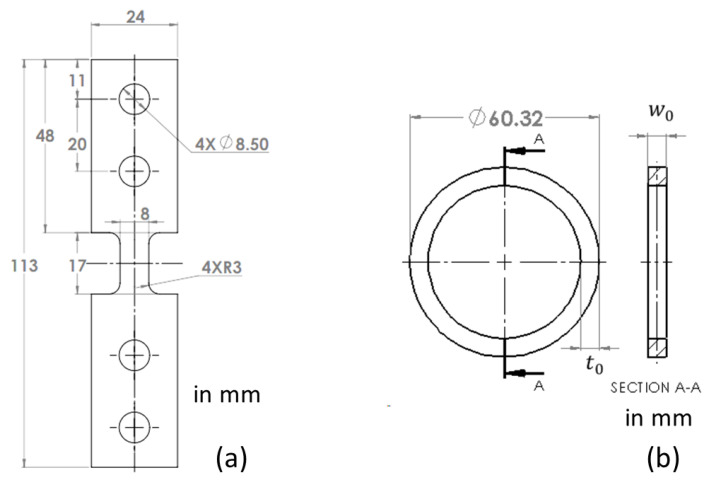
Dimensions of specimens used in the study: (**a**) dog-bone specimen and (**b**) pipe ring specimen.

Dog-bone specimens were waterjet cut from the CPVC sheets with a gauge section of 11 mm long and 8 mm wide, and the pipe ring specimens from the CPVC pipe with dimensions same as those used previously [13]. Dimensions for these two types of specimens are given in Figure 1.

Acetone and primer were chosen as the chemical solvents to treat the specimens. Acetone was ACS reagent grade of ≥99.5% from Sigma-Aldrich (St. Louis, MO, USA), purchased from the Chemical Stores at the University of Alberta. Primer used in this study is known as clear primer, purchased from McMaster-Carr which was same as the one used in the previous study [13], containing acetone of 60% by weight.

**Figure 2:**
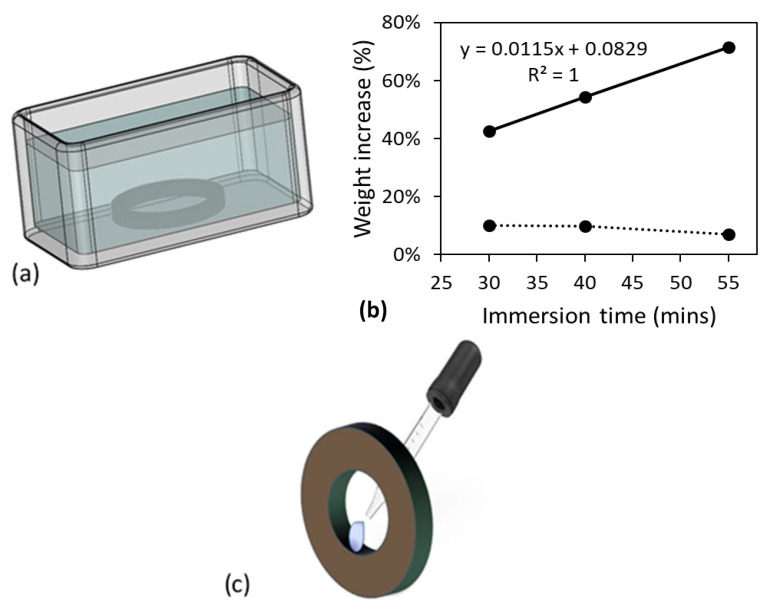
Specimen treatment methods used in this study: (**a**) setup for the immersion treatment, depicted using a pipe ring specimen, (**b**) weight gain of dog-bone specimens as a function of immersion time in acetone, and (**c**) local treatment of a pipe ring specimen on its inner surface.

Setup for the immersion treatment was same as that used previously [13], for both dog-bone and pipe ring specimens, as depicted in Figure 2a using a pipe ring specimen as an example. In the immersion treatment, the chemical solvents were replaced when tiny CPVC bits were seen in the solvents, so that the specimens were immersed in the relatively fresh chemical solvents. Since objective for the immersion treatment of dog-bone specimens was to saturate the specimens with the chemical solvents without dissolving the CPVC, the immersion time in primer and in acetone could be different. The immersion time for dog-bone specimens in acetone was determined based on Figure 2b which shows the change of weight gain as a function of the immersion time immediately after the immersion treatment (solid line) and after air dry for 6 h (dashed line). The former suggests that the weight gain increased almost linearly with the increase of immersion time, but the dashed line shows a noticeable drop of the weight gain by the increase of immersion time from 40 to 55 min. Difference of the trend lines for the weight gain suggests that after the immersion in acetone for 40 min, mass loss by dissolving CPVC into acetone outweighed the increase of the acetone absorption. Therefore, the immersion time of 40 min was chosen for the immersion of dog-bone specimens in acetone.

The same setup was used to determine the immersion time in primer. However, it was found that after immersion in primer for 15 min, the dog-bone specimens became very tacky and difficult to handle. Soon after 15 min in primer, the dog-bone specimens started showing mass loss. Therefore, the immersion in primer was decided to be 10 min. This observation also suggests that CPVC is easier to be dissolved in primer than in acetone.

For the pipe ring specimens, the immersion time was selected to be fixed at 30 min for both primer and acetone, in order to be consistent with that used in the previous study for the immersion treatment in primer [13]. The previous study has suggested that for this immersion period in primer, cross section of the specimens showed a core-shell structure, with the shell thickness of around 0.5 mm.

In addition to the immersion treatment, some pipe ring specimens were exposed to acetone or primer in a selected region on the inner surface, as depicted in Figure 2c, using a pipette to drip the chemical solvents in the selected region. Care was taken to make sure that the chemical solvents were only in contact with the specimens in the selected region, and such treatment is referred to as ‘local contact’ in this work. Total duration for the dripping process was 2.5 h for both primer and acetone. For all pipe ring specimens after either the immersion treatment or the local contact on the inner surface, the specimens were air dried for 10.5 days before the mechanical testing.

It should be noted that although the time range considered for the immersion treatment of dog-bone specimens in acetone was only from 30 to 55 min, a much wider time range was considered in a preliminary study using rectangular strips of 8 mm × 55 mm, to identify potential problems that could be encountered in the immersion treatment of thin specimens. The following phenomena were observed from the preliminary study:

(i) With the immersion time less than 30 min, the rectangular strips did not show any shape change
(ii) At the immersion time around 36 min, the rectangular strips started warping and showed wrinkles
(iii) At the immersion time around 39 min, the wrinkles disappeared, and the rectangular strips became flat again, and
(iv) At the immersion time around 2 h, corners of the rectangular strips started disappearing which was an indication that CPVC was dissolved in acetone.

It was based on the above observations that the time range from 30 to 55 min was selected to determine the immersion time in acetone for the dog-bone specimens without noticeable mass loss. Note that for the immersion treatment of dog-bone specimens in primer, 10-min immersion time was determined directly using dog-bone specimens, i.e., after the wrinkles disappeared and the specimens became flat again.

In addition to the above observations, the preliminary study also found that the rectangular strips could stick to the bottom of the container after the immersion in acetone for about 3 min. However, by gently shaking periodically the container and using a razor blade to separate the rectangular strip from the container surface, the problem of sticking to the container was avoided. This procedure was also used in the immersion treatment of dog-bone specimens in acetone or primer.

### 2.2. Mechanical Testing

All mechanical tests were conducted using Qualitest Quasar 100 universal test machine at the cross-head speed of 1 mm/min. Mechanical testing on dog-bone specimens was based on the conventional test setup for tensile test of plastics, using grips of 42 mm long. With the specimen tab length of 48 mm, as shown in Figure 1a, a gap of 6 mm was provided between the grip edge and the edge of the gauge section so that premature fracture due to stress concentration by the gripping could be avoided. For pipe ring specimens, the D-split test setup was used, same as that used previously [13] but without any extensometer attached to the gauge section. This was because the post-immersed specimens with a short drying period of less than 24 h were too soft to support the extensometer. All mechanical tests were duplicated to ensure good reproducibility of the test results.

For the dog-bone specimens treated in acetone, drying periods up to 984 h were used to examine the change of the stress-stroke curves with the drying time, and for the dog-bone specimens treated in primer, the drying periods were up to 2230 h to ensure that the weight gain as a function of drying time reached a plateau, as to be presented in the Section 3. For pipe ring specimens, all specimens were dried for the same period of 10.5 days before the mechanical testing. This drying period was chosen based on results from the previous study [13] which suggested that after the immersion treatment in primer for 30 min, pipe ring specimens with a drying period of 10.5 days gave the maximum fracture strain in the mechanical testing.

It should be noted that the air-dry process could cause warping of the dog-bone specimens. This was avoided by firstly drying the dog-bone specimens in air for the first 6 h, and then clamping the specimens using polyethylene (PE) blocks for the drying period from 6 to 23 h so that the specimens gained sufficient stiffness to resist the warping. Afterwards, the specimens were air dried without the PE blocks till the time for the mechanical testing. Air dry at elevated temperature was not used, to avoid the specimen warping that could be introduced if non-uniform temperature was introduced to the specimens.

### 2.3. Fracture Surface Examination

Fracture surfaces of pipe ring specimens, generated in the D-split tests, were examined using a Zeiss Sigma field emission scanning electron microscope (SEM), operated in a variable pressure mode using a backscattering detector. Since these SEM samples did not have any coating, chamber of the microscope was back-filled with N_2_ gas to minimize charging on the sample surface.

## 3. Results and Discussion

**Figure 3:**
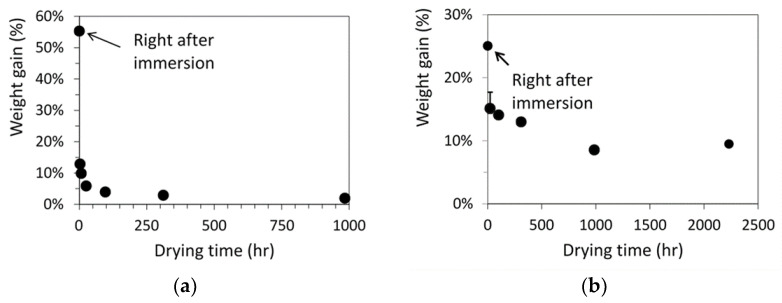
Weight gain of dog-bone specimens as a function of drying time after the immersion in (**a**) acetone for 40 min and (**b**) primer for 10 min.

Figure 3 summarizes the percentage of weight gain for the dog-bone specimens as a function of drying time after the immersion either in acetone for 40 min, Figure 3a, or in primer for 10 min, Figure 3b. As shown in these figures, initial amount of acetone absorbed in the specimens was more than twice of the amount of primer. However, after the air-dry process, the residual acetone left in the specimens was about 2 wt% of the virgin specimens, much less than the residual primer of around 9 wt%, even though the maximum drying time for the specimens immersed in acetone was less than half of the drying time for the specimens immersed in primer. Note that each point in Figure 3 represents the average value from at least three specimens. Error bars were also included in Figure 3 to indicate the range from minimum to maximum values, but the ranges were smaller than the size of the markers for the average values, except the second point from the left in Figure 3b.

**Figure 4:**
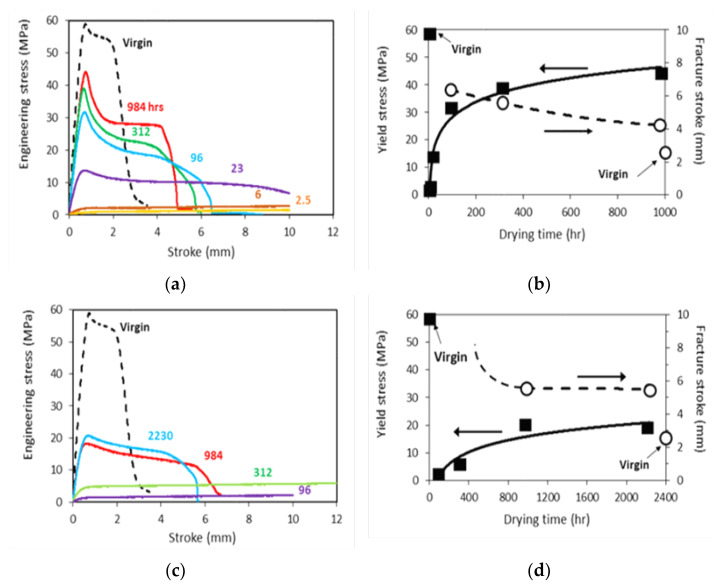
Mechanical test results for dog-bone specimens after immersion in acetone for 40 min or in primer for 10 min, and then dried for a period in hours as indicated by the numbers in (**a**,**c**): (**a**,**b**) in acetone, and (**c**,**d**) in primer.

Figure 4 summarizes mechanical test results for dog-bone specimens. Figure 4a,c present the typical engineering stress-stroke curves for specimens with different drying time (as shown by the numbers in each figure), after immersion in acetone and primer, respectively, and Figure 4b,d the corresponding average values for yield stress and fracture stroke, each from two specimens, as functions of drying time, including values for the virgin specimens as a reference. Data in Figure 4 suggest a general trend that yield stress increases and fracture stroke decreases with the increase of drying time. However, after a given air drying period, specimens immersed in acetone showed higher yield stress than specimens treated in primer, filled squares in Figure 4b,d, respectively, and both yield stress values were lower than the yield stress for virgin specimens. As shown in Figure 3, difference of the yield stresses was because both acetone and primer acted as plasticizers for CPVC. With a lower plasticizer content, drop of the yield stress for the dog-bone specimens was smaller. In addition, after air dry for 984 h, yield stress in Figure 4b was higher than the corresponding value in Figure 4d after air dry for 2230 h. This also suggests that acetone was much easier than primer to remove from CPVC. This is consistent with the change of weight gain shown in Figure 3. That is, residual acetone content was much lower than residual primer content, even though the former was after a shorter air-dry period. Unfilled circles in Figure 4b,d also suggest that ductility change in terms of stroke for the onset of fracture showed the opposite trend to that for yield stress. That is, comparing fracture stroke for specimens treated in primer after air dry of 2230 h, Figure 4d, with those treated in acetone after air dry of 984 h, Figure 4b, the former was larger than the latter even after a longer drying period, and both were larger than that for the virgin specimens (about 2.5 mm). This trend could also be explained by the plasticizer effect and the higher residual primer content than the acetone content.

In addition to the trends for yield stress and fracture stroke in Figure 4b,d, engineering stress-stroke curves shown in Figure 4a,c also indicate that with a short drying time, i.e., less than 6 h in Figure 4a and 312 h in Figure 4c for specimens immersed in acetone and primer, respectively, these curves do not show any peak stress. This suggests that these specimens deformed uniformly through the whole gauge section till the end of test without fracture, and that residual acetone and primer in the specimens were sufficient to act as a plasticizer to allow large deformation without crack initiation. Therefore, degradation was not introduced to CPVC by immersion in these chemical solvents. Note that all mechanical tests, except the curve with drying time of 312 h in Figure 4c, were stopped when the stroke reached 10 mm. Therefore, specimens after drying periods of 2.5, 6 and 23 h in Figure 4a, and 96 and 312 h in Figure 4c did not fracture at the end of the tests.

**Figure 5:**
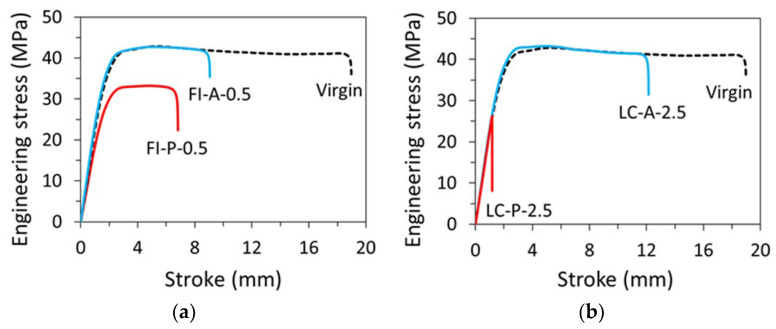
Engineering stress–stroke curves for pipe ring specimens: (**a**) after full immersion for 0.5 h in acetone (FI-A-0.5) or primer (FI-P-0.5) and then air dried for 10.5 days, and (**b**) after local contact for 2.5 h with acetone (LC-A-2.5) or primer (LC-P-2.5) and then air dried for 10.5 days.

Figure 5 presents typical engineering stress-stroke curves for pipe ring specimens after either the full immersion treatment (FI), Figure 5a, or through the local contact (LC) on the inner surface, Figure 5b. ‘A’ in the curve labels in Figure 5 stands for acetone, ‘P’ primer, and ‘0.5′ and ‘2.5′ time in hours for full immersion and local contact, respectively. For example, the curve labelled as ‘FI-P-0.5′ in Figure 5a was from a specimen after full immersion in primer for 0.5 h. Note that as mentioned earlier, all pipe ring specimens were air dried for 10.5 days prior to the mechanical testing, to be consistent with one of the drying periods used in the previous study [13]. Figure 5 also includes a reference curve (using a dashed line) which was from a virgin specimen without any treatment.

Figure 5a suggests that after the immersion-and-air dry process, the pipe ring specimens treated in primer (FI-P-0.5) had a much lower yield stress than that for the virgin specimens, consistent with the results reported previously [13]. However, for the pipe ring specimens treated in acetone and then air dried for 10.5 days, as shown by the curve FI-A-0.5 in Figure 5a, yielding could occur at the same stress level as the virgin specimens, though ductility in terms of fracture stroke was reduced. The possibility of recovery of the maximum stress to the level for the virgin specimens also suggests that acetone could easily be removed from CPVC, and that the air dry for 10.5 days was sufficient to reduce the acetone content in the pipe ring specimen to a negligible level, to avoid its effect on the yield stress of the material, though a detailed investigation using a large number of specimens would be needed to determine the critical drying time needed after the immersion treatment in acetone to reach a full recovery of the yield stress for the specimens.

The corresponding pipe ring specimens treated through local contact with acetone and then air dried for 10.5 days, as shown by LC-A-2.5 in Figure 5b, also showed the same yield stress level as that for the virgin specimens. However, the corresponding specimens treated in primer and then air dried for 10.5 days, i.e., either FI-P-0.5 in Figure 5a or LC-P-2.5 in Figure 5b, the peak stress was much lower than that for the virgin specimens. As the curve for LC-P-2.5 in Figure 5b shows a sudden drop of the engineering stress while the curve was following the path of load increase of the virgin specimen, and the specimen fractured in a brittle manner, the LC-P-2.5 specimen was fractured before yielding could be generated. Although the change from a ductile, post-yield fracture for the virgin specimens, to the brittle, prior-to-yield fracture after the treatment in primer could be interpreted as a ductile-brittle transition, it needs to be pointed out that the specimens with the ‘local-contact’ treatment could not be fully saturated with the primer on the cross section where the local contact with primer was made. Rather, primer was partially absorbed on the cross section and thus, the cross section had a two-layered structure in which only the layer close to the inner surface contained primer [13]. In view of such a two-layered structure on the cross section, rather than referring this change of fracture behavior as a ductile–brittle transition, other possible reasons for its occurrence, such as the interaction between the relatively soft, low strength inner layer and the stiff, high strength outer layer, should be considered. Only after these reasons are ruled out could we consider this phenomenon as a ductile-brittle transition. Note that both plots in Figure 5b for specimens after the local contact treatment also indicate that after the drying period of 10.5 days, initial slope of the engineering stress-stroke curves became similar to that for the virgin specimens, suggesting that after air dry of 10.5 days, the inner layer in both LC-P-2.5 and LC-A-2.5 has gained sufficient stiffness to show little effect of the residual chemical solvents on the overall specimen stiffness.

**Figure 6:**
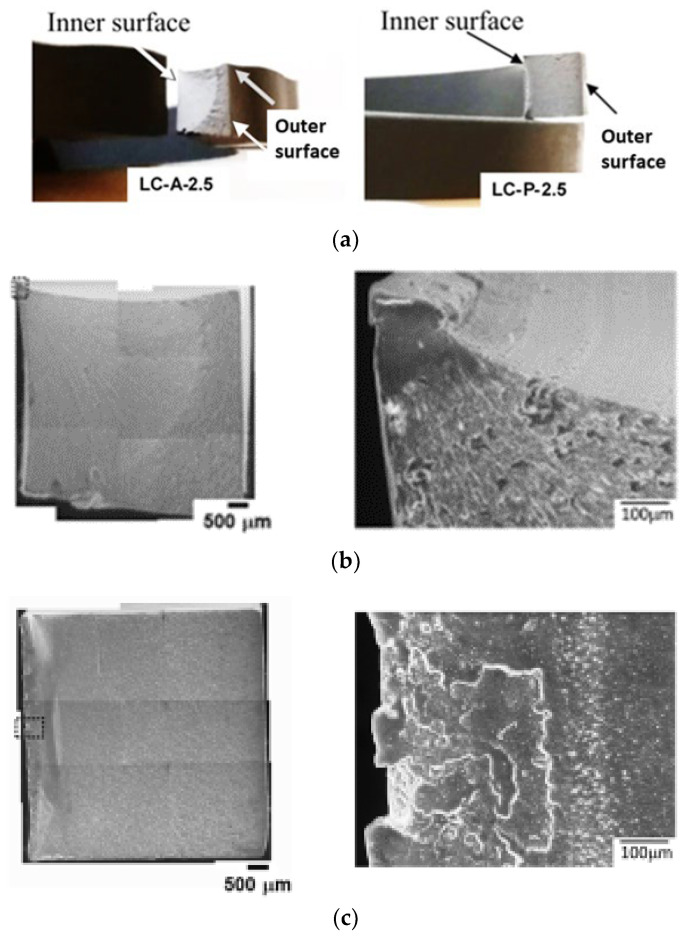
Fracture surfaces of pipe ring specimens after the treatment through local contact on the inner surfaces: (**a**) optical photographs showing the overall fracture surfaces, (**b**) SEM micrographs from an LC-A-2.5 specimen, and (**c**) SEM micrographs from an LC-P-2.5 specimen. For micrographs in (**b**,**c**), inner surface is on the left, and outer surface on the right.

To further investigate the fracture behavior of the pipe ring specimens treated through local contact with either primer or acetone on the inner surface, fracture behaviors of these specimens were inspected microscopically, as presented in Figure 6. Figure 6a presents optical photographs, showing the overall fracture surfaces before the SEM examination. The left photograph in Figure 6a was from a post-tested LC-A-2.5 specimen, showing a fan-shaped area that was generated from a corner near the inner surface, and the right photograph from LC-P-2.5, showing a narrow band along the vertical direction, generated across the edge along the inner surface.

The corresponding SEM micrographs for Figure 6a are shown in Figure 6b for LC-A-2.5, and Figure 6c for LC-P-2.5. Left micrographs in Figure 6b,c are composite micrographs, by combining many SEM micrographs at the same magnification to show the overall fracture surfaces. SEM micrographs of relatively high magnification for the regions boxed in the left composite micrographs using dash lines, are presented on the right of Figure 6b,c, depicting the flat crack initiation zones which do not show any sign of crack initiation from a dominant defect. This is an indication of ESC [14]. Since crack initiation in LC-A-2.5 started after the yielding, but that in LC-P-2.5 before, as indicated by the relative stroke position for the sudden stress drop with respect to the stroke for the maximum stress, the region where crack was initiated in the former was smooth while that in the latter consisted of multiple flat regions with slightly different elevations.

Another difference between Figure 6b,c is the extent of stable crack growth before the transition to relatively fast crack growth. The left, composite micrograph in Figure 6b shows that the crack started from a corner of the cross section near the inner surface, which grew in a stable manner to form a fan shape with area covering more than half of the cross section before the fast growth. The corresponding micrograph in Figure 6c, on the other hand, shows crack initiation along the edge adjacent to the inner surface, which led to fracture through a relatively fast crack growth, resulting in a sudden stress drop shown by the curve of LC-P-2.5 in Figure 5b. Features on the fracture surfaces to indicate ductile and brittle behaviors and the transition from stable to fast crack growth have been well documented in Ref. [15]

Note that the fan-shaped region shown in the left micrograph of Figure 6b is consistent with the observation reported previously, i.e., Figure 9a in ref. [13] for dry (virgin) specimens. This suggested that after a drying period of 10.5 days, LC-A-2.5 could fracture in a manner similar to the virgin specimens.

For LC-P-2.5, the absorption of primer through the inner surface (i.e., the left edge on the cross section of Figure 6c) resulted in a left part of the cross section with a reduced yield stress. Although Figure 4d suggests that decrease of yield stress could be accompanied by the increase of fracture ductility, the fracture surface shown in Figure 6c suggests that crack started from the region of low yield stress. Therefore, it is speculated that the high fracture ductility of the region has been suppressed by the adjacent region that has high yield stress and low fracture ductility. Growth of the crack to the adjacent region of high yield stress and low ductility resulted in the brittle fracture. Godart and Leevers [16] discussed possible fracture scenarios in a bi-layered polymer structure. However, the discussion was focused on the crack initiation in the thin, brittle layer, and crack growth into the tough substrate to result in a brittle fracture, rather than the ductile fracture for the pure substrate without the thin, brittle layer. To our knowledge, the scenario of crack initiation from the region of low yield stress and high ductility has not been investigated in the past. We speculate that ductility of the low yield-stress layer is suppressed due to its bonding to the high strength and stiff substrate. Therefore, the overall fracture behavior is brittle, as shown by the LC-P-2.5 specimens. However, such a speculation needs to be verified in a future study.

Results from the pipe ring specimens presented in Figure 6 also suggest that after the exposure to primer, the specimens show a bigger reduction in strength and ductility than those exposed to acetone. Therefore, if excessive amount of primer was applied in the solvent welding process, to result in primer on some inner surface of the pipe which was not covered by adhesives, there could be an adverse effect on the mechanical properties of the CPVC pipe. In other words, primer applied in solvent welding process could actually reduce the load-carrying capability of the CPVC pipe. Although in the bonding assembly, reduction in the mechanical performance of CPVC could be offset by the load-carrying enhancement from the matching counterpart, such an offset benefit does not exist for the CPVC pipe section that is in contact with the excessive primer and is not part of the bonding assembly. This scenario could not be avoided, especially in an environment of low temperature or in a welding process that needs a relatively long time to complete, resulting in decrease of strength and ductility of CPVC. This could lead to generation of micro-cracks from the inner surface of the pipe, especially for pipes with a relatively high residual stress [17] or made of CPVC resin of relatively low molecular weight. These micro-cracks could evolve over time to cause premature pipe failure.

## 4. Conclusions

Influence of acetone and primer on mechanical properties for CPVC sheet and pipe was investigated using dog-bone specimens from sheets and ring specimens from pipe. Results from the former indicate that absorption of acetone and primer caused decrease in yield stress of CPVC but increase in the ductility (based on the fracture stroke), and that primer was much more difficult to remove than acetone from CPVC through an air-dry process. The primer also showed a stronger effect on the decrease in yield stress, which was likely caused by the larger amount of residual primer left in the specimens even after a long drying period.

Results from the pipe ring specimens showed that decrease of yield stress, after treatment with acetone through either immersion or local contact, could be fully recovered after an air-dry period of 10.5 days. However, such a quick yield stress recovery was not observed for specimens treated with primer.

In view that ductility of thin specimens increases due to the absorption of acetone or primer, it is unlikely that these chemical solvents could cause ductility decrease of CPVC pipe by degrading its molecules. However, results from the pipe ring specimens suggest that the overall ductility decrease by the absorption of primer or acetone could be related to the two-layered structure on the cross section, of which one layer contained solvents and the other not. To our knowledge, none of the work in the literature could provide a plausible explanation for the ductility decrease of LC-P-2.5 pipe ring specimens observed in this study. Further investigation to clarify mechanisms responsible for such a ductility decrease is needed and is being planned when this paper is prepared.

## Data Availability

The data supporting the findings described in this manuscript are available from the corresponding authors upon request.
